# Specialist Psychiatric Bed Utilisation by People With Intellectual Disabilities and Autistic People: A Time‐Series Analysis Using the English Assuring Transformation Dataset

**DOI:** 10.1111/jir.70001

**Published:** 2025-06-12

**Authors:** Atiyya Nisar, Paul A. Thompson, Harm Boer, Haider Al‐Delfi, Peter E. Langdon

**Affiliations:** ^1^ Intellectual Disabilities Research Institute (IDRIS) University of Birmingham Birmingham UK; ^2^ Coventry and Warwickshire Partnership NHS Trust Coventry UK; ^3^ REACH OUT West Midlands Provider Collaborative for Adult Secure Care Birmingham UK; ^4^ Birmingham Community Healthcare NHS Foundation Trust Birmingham UK; ^5^ Herefordshire and Worcestershire Health and Care NHS Trust Worcester UK

**Keywords:** autism, inpatient, intellectual disabilities, length of stay, psychiatric beds, time series, transforming care

## Abstract

**Background:**

Using nationally available anonymised and aggregated English data, we examined specialist and nonspecialist psychiatric bed utilisation by people with intellectual disabilities and/or autism.

**Methods:**

Using data about specialist psychiatric bed utilisation from the Assuring Transformation Dataset, from March 2015 to January 2024, we applied linear regression (with moving average or autoregressive errors) to explore the relationships between a set of outcome variables (e.g., number of inpatients and length of stay) and a set of sociodemographic, clinical and service‐related predictor variables (e.g., age, ethnicity, admission source, legal status, admission source, discharge destination, Care (Education) and Treatment Reviews) over time. Comparisons were made with data from the Mental Health Services Data Set about nonspecialist psychiatric bed utilisation.

**Results:**

Over time, there was an average reduction of 8.07 inpatients per month. This reduction was due to a reduction in the number with a length of stay longer than 2 years, and fewer inpatients with intellectual disabilities without autism over time, rather than fewer autistic inpatients without intellectual disabilities; instead, the number of autistic inpatients increased by 6.02 per month. However, overall, there were fewer inpatients in specialist psychiatric beds than in nonspecialist beds by an average of 877 patients, and the number in specialist beds reduced faster than the number in nonspecialist beds over time. We found that more hospital spells were associated with more inpatients older than 18, more detentions under Part III of the Mental Health Act, more inpatients not known to the local authority, and an increased number of White inpatients. More admissions were associated with fewer discharges, while those with a hospital stay longer than 2 years were less likely to have had a postadmission Care (Education) and Treatment Reviews and were more likely to use advocacy.

**Conclusions:**

The number of inpatients with intellectual disabilities in specialist psychiatric beds continues to decline over time, while the number of autistic inpatients without intellectual disabilities is increasing. Future research should utilise participant‐level data to explore patient long‐term trajectories.

## 1 Background

Reducing psychiatric bed utilisation by individuals with intellectual disabilities and/or autistic people has been an explicit priority in England since the abuse of patients at Winterbourne View hospital in 2011 (Department of Health 2012). As a consequence of concerns about the quality of inpatient psychiatric care, NHS England (2015) developed and implemented the Transforming Care programme, which aimed to reduce psychiatric inpatient numbers by 35%–50% whilst also increasing community‐based care capacity. Transforming Care focused upon improving the quality of care offered to people with intellectual disabilities and autistic people, while also improving quality of life, preventing inappropriate psychiatric hospital admission and shortening length of hospital stay. The key mechanisms leading to these changes were investment in community‐based care and the implementation of Care (Education) and Treatment Reviews (C(E)TRs). C(E)TRs aimed, ‘to bring a person‐centred and individualised approach to ensuring that the care and treatment and differing support needs of the person and their families are met, and that barriers to progress are challenged and overcome’ (p.10; NHS England 2017). C(E)TR meetings occur when someone is at risk of admission, or has been admitted, to a psychiatric hospital. The C(E)TR is run by an independent panel comprised of an Expert by Experience (EbE), a clinician and the commissioner. The patient and their family are invited, and together with the clinical team, care is reviewed, and the panel determines whether a person's needs could be better met in a community setting with additional support.

However, the Transforming Care goal to reduce inpatient numbers by 35%–50% was not met (Langdon et al. 2023). In 2019, the government set a new target to reduce the number of inpatients by less than half the number that was in a psychiatric hospital in 2015 (Department of Health and Social Care 2019). This target was also missed, and a new target was then set, which was to reduce the number of inpatients within psychiatric hospitals by 10% during the years 2025 to 2026 (NHS England 2025). Nissar et al. [In Press] argued that one of the reasons that these targets were missed is that Transforming Care was affected by British financial austerity leading to a lack of investment in community‐based services, including services for people with intellectual disabilities (Forrester‐Jones et al. 2021).

While there is a continued focus upon reducing the number of psychiatric beds for people with intellectual disabilities and/or autistic people in England, there is evidence that inpatient psychiatric care is beneficial when it is designed to meet the specific needs of this population when such care is genuinely needed (Burrows et al. 2023; Melvin et al. 2022). The demand for inpatient psychiatric care for individuals with intellectual disabilities and/or autistic people persists, and although some of the need can be met through the provision of community‐based services, closing local beds may lead to out‐of‐area admissions, if beds are unavailable; this can present further challenges, such as delaying discharge in some cases (Abraham et al. 2022) and making it difficult for family to maintain contact. Some have argued that closing psychiatric beds for this population may lead to an increase in the number being sentenced to prison (Taylor et al. 2017), and there is evidence that psychiatric bed closure is associated with an increase in the number of prisoners being transferred into psychiatric hospitals (Keown et al. 2019), and an increase in the prison population (Wild et al. 2022).

Across the four nations of the United Kingdom, there is specialist state‐funded community and inpatient psychiatric services for people with intellectual disabilities (Perera and Courtenay 2018; Melvin et al. 2022), with some limited specialist provision for autistic people without intellectual disabilities (Melvin et al. 2022). These services have clinical staff with specialist training, enabling them to work with individuals with intellectual disabilities, with some having specialist professional registration (e.g., registered intellectual disability nurse). However, while there are specialist inpatient services for people with intellectual disabilities in the United Kingdom, it is the case that many individuals are admitted to nonspecialist psychiatric hospitals, units or wards. In these instances, care is often provided by clinical staff who lack or have limited specialist training or experience in working with people with intellectual disabilities.

It is the case that there is a lack of specialist inpatient psychiatric provision for people with intellectual disabilities and specialist professional training in working with people with intellectual disabilities in many other countries (Holt et al. 2000; Jaydeokar et al. 2020; Kwok and Chui 2008; Lunsky et al. 2007; Melvin et al. 2022). In many nations, when someone with an intellectual disability requires psychiatric admission, they are admitted to general psychiatric hospitals, units or wards, alongside those without intellectual disabilities. There is some evidence that these admissions to nonspecialist hospitals, units or wards are associated with more prescribing of psychotropic medication, increased observation and staffing, and more use of seclusion for longer periods (Lohrer et al. 2002; Melvin et al. 2022; Turner and Mooney 2016; White et al. 2010).

In England, we have access to publicly available aggregated data about specialist and nonspecialist psychiatric bed utilisation by autistic children and adults and children and adults with intellectual disabilities within two datasets published monthly by NHS Digital. The first is called the Assuring Transformation (AT) Dataset and is solely about the utilisation of specialist psychiatric beds (i.e., beds commissioned specifically for people with intellectual disabilities and/or autistic people), whilst the Mental Health Services Dataset (MHSDS) is a national dataset of all patients in contact with mental health services across England, including data regarding monthly inpatient bed utilisation for all types of psychiatric beds. Data pertaining to the number of inpatients with intellectual disabilities and/or autistic inpatients admitted to a nonspecialist psychiatric bed are included within MHSDS.

The AT data have been used previously within a time‐series modelling study for the period December 2013 to March 2021, and a 21% or 24% reduction in the number of psychiatric inpatients was reported during this time period (Langdon et al. 2023). The differences in the calculated reduction over time were because of two differing types of data within the AT dataset. Langdon et al. (2023) also identified that over time, periods where there were more consultant psychiatrists working in the National Health Service were associated with decreases in the number of hospital stays. They also reported that more pre‐admission C(E)TRs were associated with increased admissions over time, while more postadmission C(E)TRs were associated with increases in discharges, also over time.

We previously conducted a time‐series analysis using data from the MHSDS to explore what factors influence psychiatric bed utilisation by people with intellectual disabilities and autistic people in all psychiatric beds across England, including nonspecialist beds (Nisar et al. [In Press]). We found that (1) the number of hospital stays decreased on average by 4.55 per month over time, and this was mainly amongst those who had been in hospital for 2 years or longer, (2) periods when hospital stays were higher were associated with periods when there were more children and non‐White people, relative to White people, in hospital, (3) periods when admissions were higher were associated with more patients being detained under Part II of the Mental Health Act, 1983, as amended, 2007, and these patients were also more likely to be subject to restraint, and (4) periods when discharges were higher were associated with a decrease in the number of White, relative to non‐White, inpatients.

Within England and Wales, any person who meets the criteria as defined within Mental Health Act, 1983, as amended, 2007, can be detained within a psychiatric hospital using Part II of the Mental Health Act. To be detained under Part II, two medical doctors must agree, and one of these two must be authorised under s.12 of the Act; the agreement of an Approved Mental Health Practitioner or nearest relative is also a requirement. Admission to a psychiatric hospital is also possible using Part III of the Mental Health Act. However, this can only be ordered the courts, usually by a Crown Court judge, who has received evidence from two medical doctors, and is typically used to divert individuals from criminal justice into inpatient psychiatric care. In all cases of authorised detention, a patient is entitled to a psychiatric bed.

Considering the continued emphasis upon reducing psychiatric hospital bed utilisation by people with intellectual disabilities and/or autistic people in England, it is important for policymakers to establish if specific factors are linked to such utilisation, as these may influence the nature of the alternatives to inpatient care, and may provide important information to inform service design and care pathways. In the current study, we utilised data from the AT Dataset to complete a further time‐series analysis of specialist inpatient psychiatric bed utilisation, and we used data from the MHSDS to compare specialist and nonspecialist psychiatric bed utilisation by individuals with intellectual disabilities and autistic people. Our study was different from Langdon et al. (2023) in three ways: (1) we made use of more AT data as it had become available; (2) we made comparisons between specialist and nonspecialist psychiatric bed utilisation; and (3) we compared bed use over time by people with intellectual disabilities, people with intellectual disabilities and autism and people with autism only. Our further aim was to explore the relationship between various sociodemographic, clinical and service‐related predictor variables, and the following outcome variables, from the AT Dataset: (1) total monthly number of hospital spells, (2) total monthly number of discharges, (3) total monthly number of admissions, (4) number of inpatients with a length of stay under 2 years and (5) number of patients with a length of stay over 2 years.

## 2 Methods

### 2.1 Data Extraction

The data in this study were downloaded from NHS Digital (https://digital.nhs.uk/data). Specifically, we downloaded all AT data relating to specialist psychiatric bed utilisation by people with intellectual disabilities and/or autistic inpatients for the period between December 2013 and February 2024; however, some data were not available until March 2015 including the total number of inpatients. We also downloaded MHSDS data regarding monthly inpatient numbers for all psychiatric beds for the period between March 2018 and January 2024 (see Nisar et al., [In Press] for further detail regarding this data). Outcome and predictor data were extracted as either frequency counts or were converted to ratios representing the proportion of individuals with a particular characteristic (Table 1 provides interpretations for the predictor variables converted to ratios).

**TABLE 1:** Interpretation of predictor variables converted to ratios.

Predictor variable	Interpretation
Ethnicity ratio	Values > 1 indicate more White inpatients relative to non‐White discharges
Planned admission ratio	Values > 1 indicate more planned admissions relative to unplanned admissions
Autism to intellectual disabilities ratio	Values > 1 indicate more autistic inpatients relative to inpatients with intellectual disabilities
Ward security ratio	Values > 1 indicate more inpatients in forensic wards relative to inpatients in acute wards
Legal status ratio	Values > 1 indicate more patients detained under Part II of the Mental Health Act relative to those detained under Part III of the Mental Health Act
Advocacy ratio	Values > 1 indicate more patients using advocacy relative to those not using advocacy
Pre‐admission C(E)TR ratio	Values > 1 indicate more inpatients with a Pre‐Admission C(E)TR relative to those without
Postadmission C(E)TR ratio	Values > 1 indicate more inpatients with a Postadmission C(E)TR relative to those without
Local authority aware	Values > 1 indicate local authority awareness relative to the local authority not being aware
Discharge destination ratio	Values > 1 indicate more patients discharged to the community relative to those discharged to another hospital

The outcome variables in the analysis were: (a) Hospital Spells: total monthly number of hospital spells open at the end of the month (this is defined as a spell as an inpatient within a hospital), (b) Hospital Admissions: total monthly number of hospital admissions, (c) Hospital Discharges: total monthly number of hospital discharges, (d) Length of Stay—Under 2 Years: total number of patients with a length of stay under 2 years and (e) Length of Stay—Over 2 Years: total number of patients with a length of stay over 2 years.

The predictor variables in the analysis were: (a) Age—Under 18: total monthly number of inpatients under the age of 18, (b) Age—Over 18: total monthly inpatients over the age of 18, (c) Ethnicity Ratio: ratio of White to non‐White inpatients, (d) Source of Admission—Hospital: inpatients where the source of admission was another hospital, (e) Source of Admission—Community: inpatients where the source of admission was the community, (f) Planned Admission Ratio: ratio of inpatients with planned admissions to those with unplanned admissions, (g) Autism to intellectual disabilities ratio: ratio of autistic inpatients to inpatients with intellectual disabilities, (h) Ward Security Ratio: ratio of inpatients in forensic wards to inpatients in acute wards, (g) Legal Status Ratio: ratio of monthly informal inpatients with Legal Status—Part II (those detained under Part II of the Mental Health Act) to Legal status—Part III (total number of monthly inpatients detained under Part III of the Mental Health Act), (h) Advocacy Ratio: ratio of inpatients who used an advocate to patients who did not use an advocate, (i) Pre‐Admission C(E)TR Ratio: the ratio of inpatients with a pre‐admission C(E)TR to those without, (j) Postadmission C(E)TR Ratio: the ratio of inpatients with a postadmission C(E)TR to those without, (k) Local Authority Aware Ratio: ratio of inpatients that the local authority is aware of to those they are not aware and (l) Discharge Destination Ratio: the ratio of inpatients discharged to the community to those discharged to another hospital. An explanation of each of our chosen ratios is found in Table 1.

### 2.2 Statistical Analysis

Linear regression was fitted using generalised least‐squares to account for the dependency between sequential observations. The model error structures were specified as either moving average (MA) or autoregressive (AR) errors and diagnosed using autocorrelation (ACF) and partial autocorrelation plots (pACF) (see Table S1 for linear regression output). Specifically, AR processes regress the outcome on its own lagged values, whereas MA process is a linear combination of previous error terms.

## 3 Results

Descriptive statistics for our outcome and predictor variables are found in Tables 2 and 3, respectively. Over time, the mean of the monthly counts of inpatients, across the 11 years where data were available, indicated that a majority, 73%, of inpatients had a diagnosis of an intellectual disability or a diagnosis of both intellectual disability and autism, while a minority, 27%, only had a diagnosis of autism (Table 3).

**TABLE 2:** Descriptive statistics for outcome variables.

Outcome	N	Mean (SD)	Min	Max	Skew
Hospital spells	106	2461.13 (259.93)	2030.00	2865.00	−0.07
Hospital admissions	109	100.06 (31.32)	30.00	175.00	0.45
Hospital discharges	97	146.67 (25.14)	100.00	210.00	0.33
Length of stay—under 2 years	103	958.42 (65.06)	805.00	1095.00	−0.21
Length of stay—over 2 years	103	1332.14 (183.63)	1080.00	1665.00	0.50

**TABLE 3:** Descriptive statistics.

Predictor	N	Mean (SD)	Min	Max	Skew
Age—under 18	109	207.75 (43.52)	50.00	290.00	−0.95
Age—over 18	107	2084.11 (209.51)	1630.00	2485.00	0.41
Ethnicity (ratio)^a^	108	6.84 (0.42)	5.83	7.76	−0.19
Source of admission—hospital	108	1607.56 (134.16)	1150.00	1820.00	−0.34
Source of admission—community	108	560.47 (63.69)	400.00	660.00	−0.29
Planned admission (ratio)^b^	97	1.72 (0.31)	1.30	2.68	0.82
Autism to intellectual disabilities (ratio)^c^	108	1.18 (0.44)	0.57	2.03	0.38
Intellectual disabilities	108	1084.05 (278.11)	670	1570.00	0.33
Intellectual disabilities and autism	108	543.71 (53.53)	400	635	−0.41
Autism	108	616.06 (196.48)	190	1000.00	0.38
Ward security (ratio)^d^	108	0.92 (0.12)	0.71	1.21	0.57
Legal status (ratio)^e^	108	1.34 (0.07)	1.22	1.53	0.92
Advocacy (ratio)^f^	75	5.75 (4.49)	2.68	29.45	2.84
Pre‐admission C(E)TR (ratio)^g^	97	0.39 (0.16)	0.14	0.79	0.67
Postadmission C(E)TR (ratio)^h^	97	1.81 (4.60)	0.00	20.00	2.77
Local authority aware (ratio)^i^	108	1.43 (0.38)	0.83	2.31	0.14
Discharge destination (ratio)^j^	97	4.20 (1.49)	2.00	10.00	1.37

^a^ Values > 1 indicate more White inpatients relative to non‐White discharges.

^b^ Values > 1 indicate more planned admissions relative to unplanned admissions.

^c^ Values > 1 indicate more autistic inpatients relative to inpatients with intellectual disabilities.

^d^ Values > 1 indicate more inpatients in forensic wards relative to inpatients in acute wards.

^e^ Values > 1 indicate more patients detained under Part II of the Mental Health Act relative to those detained under Part III of the Mental Health Act.

^f^ Values > 1 indicate more patients using advocacy relative to those not using advocacy.

^g^ Values > 1 indicate more inpatients with a Pre‐Admission C(E)TR relative to those without.

^h^ Values > 1 indicate more inpatients with a Postadmission C(E)TR relative to those without.

^i^ Values > 1 indicate local authority awareness relative to the local authority not being aware.

^j^ Values > 1 indicate more patients discharged to the community relative to those discharged to another hospital.

### 3.1 Hospital Spells

**FIGURES 1 to 3:**
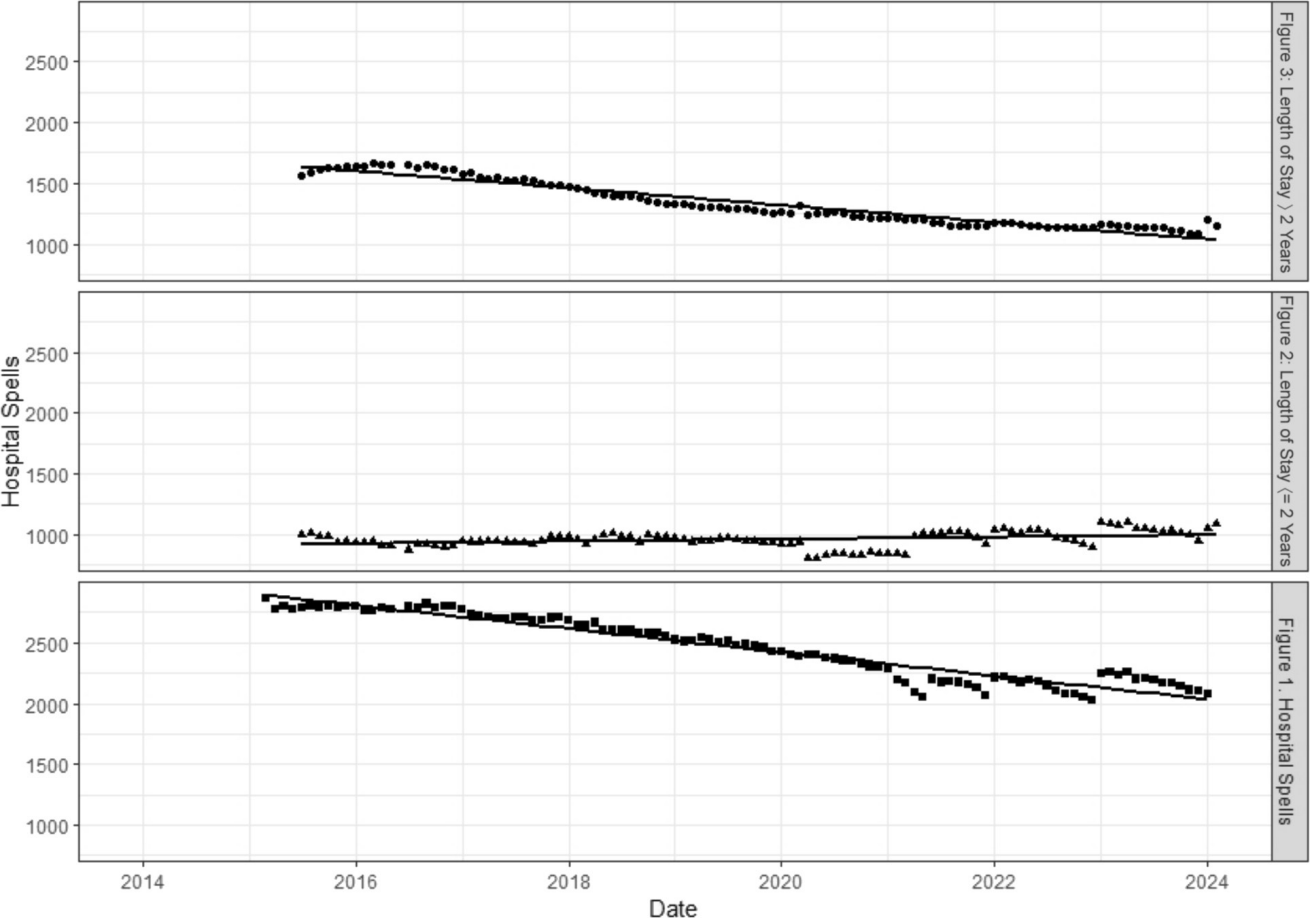
Monthly number of hospital spells, length of stay over 2 years and length of stay under 2 years over time.

For all inpatients, the number of hospital spells reduced over time. There was an average reduction of 8.07 inpatients per month, with an intercept value of 2932.45, *R* ^2^ = 0.92, Figure 1. The number of inpatients with a length of stay under 2 years remained relatively stable over time, Figure 1, with a slope value of 0.7 representing a very marginal increase but likely driven by variation in the data, and an intercept value of 912.78, *R* ^2^ = 0.11, Figure 1. For inpatients with a length of stay over 2 years, there was a reduction of an average of 5.87 patients per month with an intercept value of 1689.67, *R* ^2^ = 0.92, Figure 1 to 3.

**FIGURE 4:**
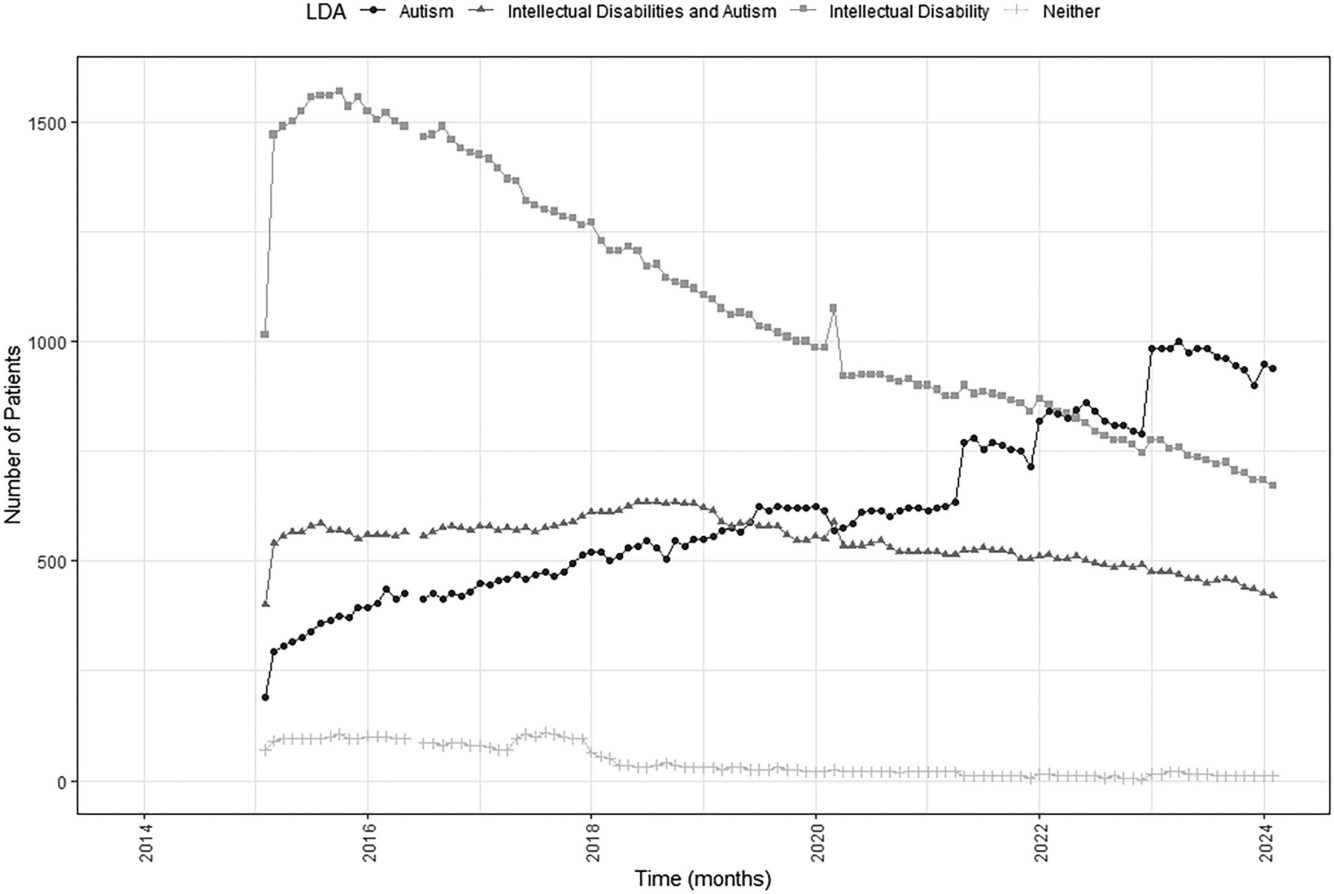
Monthly number of hospital spells for people with intellectual disabilities, intellectual disabilities and autism, or only autism.

We also examined hospital spells over time for inpatients with intellectual disabilities, those with intellectual disabilities and autism and those with only autism, separately, Figure 4. There was an average monthly reduction of 8.52 inpatients with only intellectual disabilities over time, with an intercept of 1589.99, *R* ^2^ = 0.93, and an average monthly increase of 6.02 inpatients with autism only over time, with an intercept of 258.32, *R* ^2^ = 0.94. For those with both intellectual disabilities and autism, the trend over time was best represented using piecewise linear regression with two linear segments of differing slopes. Data up to August 2018 approximated a positive linear trend, with an average monthly increase of 1.98 inpatients, while data from August 2018 approximated a negative linear trend, with an average monthly decrease of 2.68 inpatients with an intercept of 523.77, *R* ^2^ = 0.87. Across time, there was a very small number of inpatients within a specialist bed who had neither autism nor intellectual disabilities, Figure 4.

We also compared the number of inpatients in specialist psychiatric beds using data from the AT Dataset with the number of inpatients in all psychiatric beds using data from the MHSDS, Table 4.

**TABLE 4:** Linear model of the relationship between time and total inpatients within the Assuring Transformation Dataset and the Mental Health Services Dataset.

	Estimate [*Std. error*]
(Intercept)	3011.14*** [28.48]
Month	*** [0.378]
Total patients	876.85*** [71.41]
Month × total patients	3.35*** [0.86]

***p < 0.001.

**FIGURE 5:**
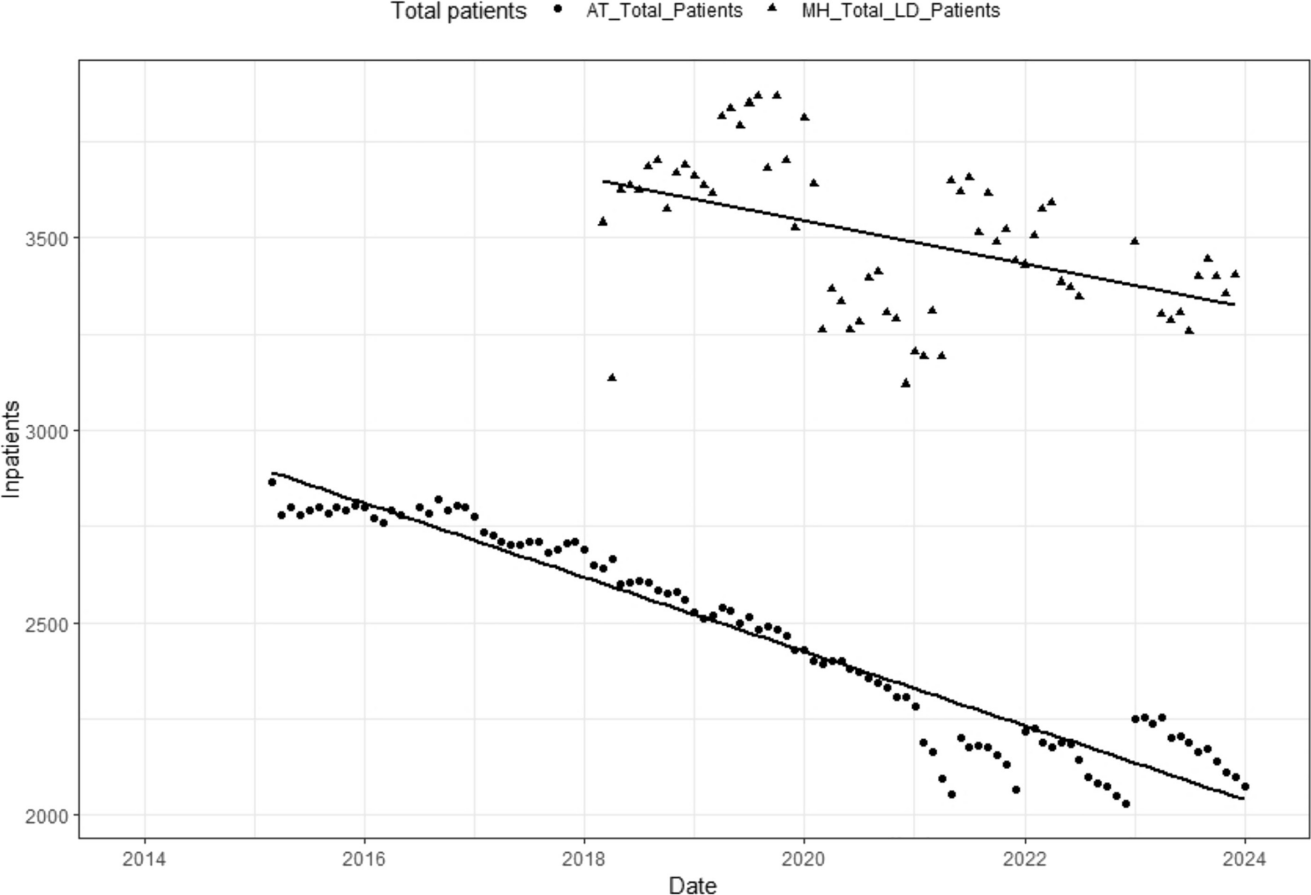
Monthly numbers of inpatients within the Assuring Transformation Dataset and the Mental Health Services Dataset.

These findings indicated that on average, psychiatric bed utilisation by individuals with intellectual disabilities and/or autistic people was higher within the data from MHSDS, relative to specialist psychiatric bed utilisation, reported within the AT Dataset, by 877 patients. There was also a significant interaction between time and dataset, with the numbers of inpatients within the MHSDS reducing at a slightly slower rate than the number of inpatients using specialist beds for people with intellectual disabilities and/or autism within the AT Dataset, Figure 5.

Examining the relationship between hospital spells for all inpatients and our chosen predictor variables revealed that periods where the number of hospital spells was greater was significantly associated with: (1) fewer inpatients aged under 18 years, *p* < 0.05; (2) more inpatients detained under Part III relative to those detained under Part II, *p* < 0.01, indicating that there were more inpatients who had been sent to hospital by the courts relative to those who are not; (3) more inpatients unknown to the local authority, relative to those known to the local authority, *p* < 0.05; (4) more White inpatients relative to non‐White inpatients, *p* < 0.01; and (5) more admissions from other hospitals, *p* < 0.05, suggesting that some inpatients are moving around the hospital system, rather than being discharged into the community, Table 5. The ratio of autistic inpatients to inpatients with intellectual disabilities was not significantly related to the number of hospital spells over time, *p >* 0.05, Table 5.

**TABLE 5:** AR models of the relationship between various predictor variables and outcome variables within the Assuring Transformation Dataset.

	Hospital spells	Admissions	Discharges	Length of stay—under 2 years	Length of stay—over 2 years
	Estimate [*95% CI*]	Estimate [*95% CI*]	Estimate [*95% CI*]	Estimate [*95% CI*]	Estimate [*95% CI*]
(Intercept)	**1448.22** *	212.55	449.60	64.81	325.53
	[37.57, 2858.87]	[−352.81, 777.90]	[−184.94, 1084.14]	[−781.00, 910.61]	[−187.28, 838.34]
Age—under 18	**−1.44** *	0.43	0.36	**1.42** ***	−0.34
	[−2.59, −0.30]	[−0.01, 0.88]	[−0.15, 0.87]	[0.75, 2.09]	[−0.75, 0.08]
Age—over 18	−0.16	0.07	0.21	0.35	0.19
	[−0.75, 0.43]	[−0.34, 0.48]	[−0.29, 0.72]	[−0.24, 0.94]	[−0.03, 0.40]
Ethnicity (ratio)^a^	**66.33** **	−17.66	−20.95	**−42.97** **	11.26
	[20.38, 112.29]	[−37.90, 2.59]	[−44.32, 2.41]	[−72.95, −12.99]	[−5.45, 27.96]
Source of admission—hospital	**1.38** *	−0.16	−0.12	−0.11	**0.44** *
	[0.33, 2.42]	[−0.59, 0.28]	[−0.64, 0.40]	[−0.75, 0.52]	[0.06, 0.82]
Source of admission—Community	0.41	0.09	−0.35	0.29	0.10
	[−0.49, 1.30]	[−0.45, 0.63]	[−1.00, 0.29]	[−0.49, 1.08]	[−0.23, 0.42]
Planned admission (ratio)^b^	11.80	2.58	−13.16	74.22	−17.62
	[−163.63, 187.22]	[−78.22, 83.37]	[−114.91, 88.58]	[−42.48, 190.92]	[−81.40, 46.15]
Autism to intellectual disabilities (ratio)^c^	−242.44	−27.45	−8.72	77.43	−135.47
	[−615.95, 131.07]	[−189.21, 134.32]	[−195.00, 177.57]	[−163.33, 318.18]	[−271.25, 0.31]
Ward Security (Ratio)^d^	−191.44	−24.51	−224.75	−112.45	19.18
	[−715.57, 332.70]	[−227.22, 178.21]	[−447.56, −1.94]	[−418.88, 193.99]	[−171.36, 209.71]
Legal Status (Ratio)^e^	**−543.95** **	20.36	−42.79	66.83	−59.81
	[−934.66, −153.23]	[−212.44, 253.16]	[−303.52, 217.94]	[−277.93, 411.58]	[−201.85, 82.22]
Advocacy (Ratio)^f^	2.76	−4.56	5.88	−12.32	**20.54** **
	[−28.98, 34.50]	[−18.52, 9.40]	[−9.52, 21.29]	[−33.33, 8.69]	[9.00, 32.08]
Pre‐admission C(E)TR (Ratio)^g^	6.25	2.97	−18.12	−1.18	16.11
	[−47.75, 60.25]	[−26.80, 32.74]	[−56.25, 20.01]	[−43.69, 41.33]	[−3.52, 35.74]
Postadmission C(E)TR (Ratio)^h^	−28.47	17.77	−0.02	−37.14	**−30.45** *
	[−102.16, 45.22]	[−36.06, 71.60]	[−72.16, 72.12]	[−111.77, 37.50]	[−57.24, −3.66]
Local Authority Aware (Ratio)^i^	**−88.57** *	15.46	−8.16	13.71	14.83
	[−154.37, −22.77]	[−11.17, 42.10]	[−37.40, 21.09]	[−26.59, 54.01]	[−9.09, 38.75]
Discharge Destination (Ratio)^j^	−5.47	**−4.44** **	−2.22	0.06	−0.23
	[−11.23, 0.29]	[−7.67, −1.21]	[−6.51, 2.08]	[−4.45, 4.58]	[−2.32, 1.86]
N	64	64	64	64	64
AIC	555.97	472.21	499.15	507.53	456.80

^a^ Values > 1 indicate more White inpatients relative to non‐White discharges.

^b^ Values > 1 indicate more planned admissions relative to unplanned admissions.

^c^ Values > 1 indicate more autistic inpatients relative to inpatients with intellectual disabilities.

^d^ Values > 1 indicate more inpatients in forensic wards relative to inpatients in acute wards.

^e^ Values > 1 indicate more patients detained under Part II of the Mental Health Act relative to those detained under Part III of the Mental Health Act.

^f^ Values > 1 indicate more patients using advocacy relative to those not using advocacy.

^g^ Values > 1 indicate more inpatients with a Pre‐Admission C(E)TR relative to those without.

^h^ Values > 1 indicate more inpatients with a Postadmission C(E)TR relative to those without.

^i^ Values > 1 indicate local authority awareness relative to the local authority not being aware.

^j^ Values > 1 indicate more patients discharged to the community relative to those discharged to another hospital.

* *p* < 0.05.

** *p* < 0.01.

*** *p* < 0.001.

### 3.2 Hospital Admissions

**FIGURE 6:**
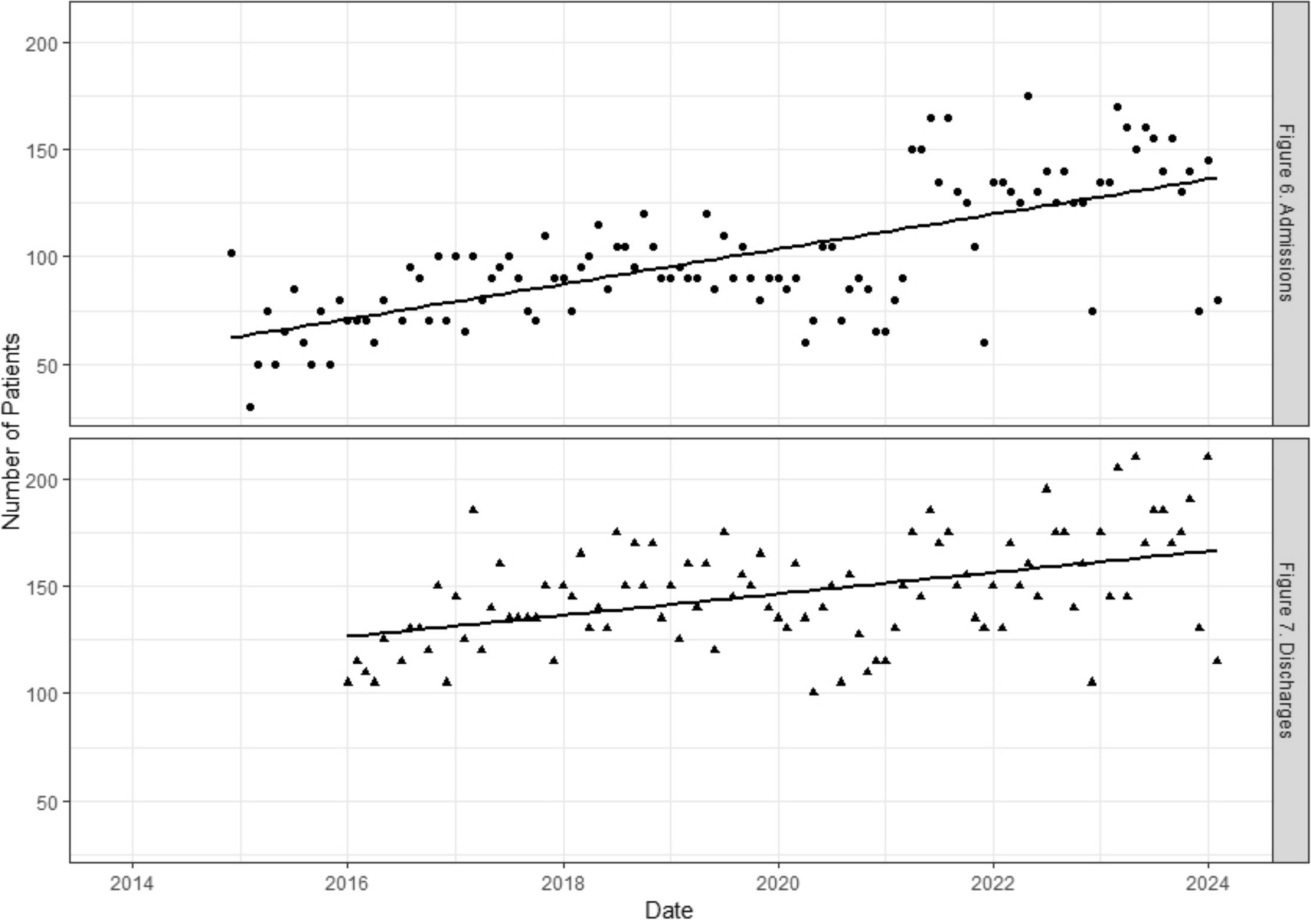
Monthly number of hospital admissions and hospital discharges over time.

The number of hospital admissions remained stable over time, Figure 4, with a slope value of 0.68 and an intercept value of 60.86, *R* ^2^ = 0.47. The number of hospital discharges were fewer, relative to the number of admissions, but also remained stable over time, Figure 6, with a slope value of 0.4 and an intercept value of 120.10, *R* ^2^ = 0.21.

Periods of time when hospital admissions were greater were associated with a decrease in the number of inpatients discharged to the community, relative to the number of inpatients discharged and admitted to another hospital, *p* < 0.05, Table 5. The ratio of autistic inpatients to inpatients with intellectual disabilities was not significantly related to admissions over time, *p* > 0.05, Table 5.

### 3.3 Hospital Discharges

There were no associations between hospital discharges and any of the predictor variables including the ratio of autistic inpatients to inpatients with intellectual disabilities, Table 5.

### 3.4 Length of Stay (Under 2 Years)

Periods of time when the number of inpatients with a length of stay under 2 years were higher was significantly associated with an increase in the number of inpatients under 18, *p* < 0.001, and a decrease in the number of White inpatients relative to non‐White inpatients, *p* < 0.01, Table 5.

### 3.5 Length of Stay (Over 2 Years)

Periods where the number of inpatients with a length of stay over 2 years was higher were associated with an increase in the number admitted from another hospital, *p* < 0.05, and an increase in the number of inpatients using advocacy, relative to those not using advocacy, *p* < 0.01. The increase in the number of inpatients with stays over 2 years was also associated with an increase in the number of inpatients who did not have a postadmission C(E)TR, relative to those who had received one, *p* < 0.05, Table 5. The autism to intellectual disabilities inpatient ratio was not related to length of stay under or over 2 years, *p > 0.05*, Table 5.

## 4 Discussion

The aim of this study was to conduct a time‐series analysis to explore how various predictor variables from the AT Dataset were related to psychiatric bed utilisation in England by people with intellectual disabilities and/or autistic people. We found that over time, the number of inpatients within psychiatric hospitals in England reduced. The difference between the first and last data point within the AT Dataset indicated a 27.6% reduction in the number of inpatients and is a slight increase over the 24% reported previously by Langdon et al. (2023) who made use of fewer data over time. The reduction in the number of inpatients was predominantly due to a reduction in the number of inpatients with a length of stay greater than 2 years and a reduction in the number of inpatients with intellectual disabilities who were not diagnosed with autism.

While the number of inpatients overall reduced, the number of autistic inpatients without intellectual disabilities increased over time. The reason for this increase is not entirely clear and could be accounted for by the substantial increase in diagnostic rates in the United Kingdom (Russell et al. 2022). Further, it is unclear from the aggregated data within the AT Dataset whether autistic people without intellectual disabilities are being admitted to existing specialist beds for those with intellectual disabilities, or specialist beds for only autistic people that have been more recently commissioned. Historically, this group would have been more likely to have been admitted to nonspecialist psychiatric beds, rather than specialist beds, and the increase in autistic people without intellectual disabilities within the AT Dataset may be accounted for by the commissioning of newer specialist beds, as well as increasing diagnostic rates over time, including amongst psychiatric inpatients (Tromans et al. 2018).

The situation over time for autistic people with intellectual disabilities was different from those with intellectual disabilities without autism and from those with autism without intellectual disabilities. The number of autistic people with intellectual disabilities within inpatient psychiatric beds was best represented by piecewise linear regression with two lines of differing slopes. The reason for an increase in numbers up to August 2018, and the subsequent decrease from August 2018, is unclear. However, it may be due to increased autism diagnostic rates amongst people with intellectual disabilities who had been in hospital for longer than 2 years, who were then subsequently discharged, leading to an eventual decrease. Unfortunately, due to the nature of the data within the AT Dataset, we were unable to examine this possibility.

We also made comparisons between the AT Dataset and MHSDS and identified that the rate of decline in the number of psychiatric beds was higher amongst specialist beds within the AT Dataset, relative to those found within the MHSDS. We also found, using the AT Dataset, that periods where there were more inpatients were associated with an increase in the number of inpatients sent to hospital by the courts, White inpatients relative to non‐White inpatients, admissions from other hospitals, and a decrease in those known to the local authority (i.e., social services) and fewer inpatients younger than 18‐years old. However, periods where there was an increase in the number with a length of stay less than 2 years were associated with more inpatients younger than 18, and a decrease in the number of White inpatients, relative to the number of non‐White inpatients. We completed a similar time‐series analysis using the MHSDS previously, and some of our findings were different. For example, overall, periods of time when the number of inpatients was higher within the MHSDS was associated with more, rather than fewer, inpatients under the age of 18 years, and an increase in the number of non‐White inpatients relative to White inpatients, rather than an increase in the number of White inpatients, relative to non‐White inpatients (Nisar et al., [In Press]). This suggests that types of beds are being utilised differently according to age and race.

Considering inpatient legal status, our current findings indicated that periods with more inpatients were associated with more inpatients detained under Part III of the Mental Health Act, 1983, as amended, 2007. In other words, an increase in the number of inpatients being sent to hospital by the courts. This finding is also different from what we observed using the MHSDS, where periods with more inpatients were related to more informal inpatients, as well as those detained under Part II and Part III of the Mental Health Act; increased admissions were associated with more detentions under Part II. These findings suggested that those sent to hospital by the courts are more likely to end up within a specialist bed, while those detained under Part II of the Mental Health Act are more likely to be admitted to a nonspecialist bed. This does suggest that nonspecialist beds are being used more during crises. However, as the programme of psychiatric bed closure in England continues, it will likely be the case that diversion for vulnerable individuals away from criminal justice will increasingly become unavailable to the judiciary, and those who would typically be detained under Part III may have to be imprisoned if no bed is available. There is evidence to suggest that this has been the case previously (Wild et al. 2022).

Periods where there was an increased number of inpatients with a length of stay longer than two years were associated with more inpatients having been admitted from another hospital, more having made use of advocacy relative to those who have not, and more inpatients having not received a postadmission C(E)TR relative to those who have received one. It is likely that those inpatients with a longer length of stay are more likely to be more complex and present with greater risk, which would explain an increased probability of being admitted from another hospital. Similarly, those with longer stays are inherently more likely to have made use of advocacy services. The relationship with C(E)TRs and the number of inpatients with a longer length of stay is in the predicted direction, but it is not possible, considering the nature of the data used within this study to determine whether there is a causal relationship between postadmission C(E)TR utilisation and the number of inpatients with a longer length of stay. Notably, Langdon et al. (2023) reported that postadmission C(E)TRs were associated with periods of time when the number of monthly discharges was higher, while they also reported that the number of pre‐admission C(E)TRs was positively related to the number of hospital admissions, but again, these relationships were not determined to be causal.

### 4.1 Clinical Implications

Utilisation of specialist psychiatric beds in England by individuals with intellectual disabilities and/or autistic people was found to be related to several factors in this time‐series analysis, which included inpatient age, ethnicity, legal status, source of admission and whether they had a postadmission C(E)TR. When considered in relation to the findings of the MHSDS time‐series analysis, inpatients under the age of 18, non‐White inpatients and those detained under Part II of the Mental Health Act are being admitted to nonspecialist psychiatric beds, rather than specialist beds specifically commissioned for people with intellectual disabilities and/or autistic people (Nisar et al., [In Press]). We found that periods when the numbers within special beds were higher were related to the number ordered to hospital by the courts. Reductions in specialist psychiatric beds may lead to increases in admissions to nonspecialist psychiatric beds, and an increase in the number sent to prison, who would have otherwise been diverted away from prison and into a specialist bed. We also noted some differences in specialist and nonspecialist psychiatric bed utilisation related to both age and ethnicity which needs to be further understood and addressed.

Transforming Care has now ended, and the bed closure goals were not achieved. The aim of the programme was to reduce the number of inpatients by investing in community‐based care and through the implementation of C(E)TRs (NHS England 2015). While we were able to include data about C(E)TRs within our models, we did not include data about community‐based care. Nevertheless, we did find that periods when admissions were higher were associated with fewer community discharges, and periods where the number of inpatients was greater were associated with more inpatients who were unknown to the local authority (i.e., social services) suggesting a relationship between community services and the number of inpatients. It remains likely that improved community mental health services for people with intellectual disabilities and/or autism are likely to be beneficial in reducing the probability of admission, and for those who are admitted, reduce the time to successful discharge. There is some evidence that enhanced community‐based care can be as effective as inpatient care for people with intellectual disabilities (van Minnen et al. 1997), and more recent evidence that intensive support teams are seen as key to helping reduce psychiatric hospital admission for people with intellectual disabilities (Hassiotis et al. 2020, 2021, 2022; Kouroupa et al. 2023).

It is also important to note that the increase in the number of autistic inpatients who do not have intellectual disabilities within specialist beds should be further examined; it may be the case that specialist beds for people with intellectual disabilities are being replaced by specialist beds for autistic people who do not have intellectual disabilities and the implications need consideration.

### 4.2 Strengths, Weaknesses and Future Research

This study extended the previous work of Langdon et al. (2023) and made use of more data points and included variables that were not included in the previous analysis such as age and ethnicity. We were also able to make comparisons with a recent analysis of nonspecialist psychiatric bed utilisation in England by this population (Nisar et al. [In Press]) and were able to make comparisons between people with intellectual disabilities, people with intellectual disabilities and autism and autistic people without intellectual disabilities.

There are some weaknesses associated with the analysis that need to be considered. We utilised observation data, and all relationships are correlational in nature; we cannot infer cause and effect. Also, we were constrained as to how many predictors were able to be included in the AR model by the number of observations. An increased number of observations would have allowed us to build more complex models. Missing data was problematic in some cases (e.g., advocacy and postadmission C(E)TR ratio). Further, we did not undertake an analysis by English region; for example, at a county or wider regional area, there may be geographical differences between different regions.

The data we used were anonymised and aggregated, which meant that we were unable to undertake further analyses comparing inpatients with intellectual disabilities, those with both intellectual disabilities and autism and autistic inpatients without intellectual disabilities using our chosen predictor variables. The authors of future studies should therefore consider making use of available national patient‐level data to explore the long‐term trajectories of inpatients with intellectual disabilities and autistic people admitted to psychiatric beds in England (both specialist and nonspecialist). This would allow for more robust modelling investigating care pathways and outcomes.

## Ethics Statement

Ethical approval was obtained through the University of Warwick Humanities & Social Sciences Research Ethics Committee (HSSREC 124/23‐24).

## Conflicts of Interest

The authors declare no conflicts of interest.

## Supporting information


**Table S1.** Linear models of the relationship between various predictor variables and outcome variables within the Assuring Transformation dataset.

## Data Availability

The data that support the findings of this study are available on reasonable request from the corresponding author.
