# Supplementary material for: Specialist Psychiatric Bed Utilisation by People With Intellectual Disabilities and Autistic People: A Time‐Series Analysis Using the English Assuring Transformation Dataset
Source: J Intellect Disabil Res. 2025 Jun 12;69(11):1224–36. doi: 10.1111/jir.70001 (PMC12576362; doi:10.1111/jir.70001)
Supplement: Supplementary file 1 — Table S1. Linear models of the relationship between various predictor variables and outcome variables within the Assuring Transformation dataset. [file JIR-69-1224-s001.docx]

| S1. Linear models of the relationship between various predictor variables and outcome variables within the Assuring Transformation dataset | | | | | |
| --- | --- | --- | --- | --- | --- |
|  | Hospital Spells | Admissions | Discharges | Length of Stay – Under 2 Years | Length of Stay – Under 2 Years |
|  | Estimate [*95% CI*] | Estimate [*95% CI*] | Estimate [*95% CI*] | Estimate [*95% CI*] | Estimate [*95% CI*] |
| (Intercept) | **1589.62 *** | 127.14 | 536.38 | -161.59 | **582.40 *** |
|  | [233.92,2945.33] | [-405.42,659.71] | [-163.00,1235.77] | [-936.35,613.17] | [124.81,1040.00] |
| Age – Under 18 | -1.00 | **0.46 *** | 0.38 | **1.64 ***** | **-0.59 **** |
|  | [-2.09,0.08] | [0.03,0.89] | [-0.18,0.94] | [1.02,2.26] | [-0.95,-0.22] |
| Age – Over 18 | -0.58 | 0.06 | 0.24 | 0.58 | 0.13 |
|  | [-1.61,0.46] | [-0.35,0.47] | [-0.29,0.78] | [-0.01,1.18] | [-0.22,0.48] |
| Ethnicity (Ratio) | **58.73 *** | -12.22 | -23.10 | **-39.62 **** | 10.81 |
|  | [9.25,108.20] | [-31.66,7.21] | [-48.62,2.43] | [-67.89,-11.35] | [-5.89,27.51] |
| Source of Admission - Hospital | **1.45 **** | -0.18 | -0.13 | -0.24 | 0.30 |
|  | [0.37,2.53] | [-0.60,0.25] | [-0.69,0.42] | [-0.86,0.37] | [-0.06,0.67] |
| Source of Admission - Community | 0.53 | 0.11 | -0.40 | 0.06 | 0.24 |
|  | [-0.82,1.87] | [-0.41,0.64] | [-1.10,0.29] | [-0.71,0.83] | [-0.22,0.69] |
| Planned Admission (Ratio) | 49.42 | 11.02 | -22.77 | 72.61 | -26.02 |
|  | [-158.35,257.19] | [-70.60,92.64] | [-129.95,84.42] | [-46.12,191.35] | [-96.15,44.11] |
| Autism to LD (Ratio) | **-590.79 **** | -27.26 | -12.09 | 174.64 | **-264.73 ***** |
|  | [-984.34,-197.24] | [-181.86,127.34] | [-215.11,190.93] | [-50.26,399.55] | [-397.56,-131.89] |
| Ward Security (Ratio) | 28.16 | 9.57 | -244.99 | -140.83 | 99.46 |
|  | [-451.37,507.68] | [-178.80,197.94] | [-492.37,2.39] | [-414.87,133.20] | [-62.40,261.32] |
| Legal Status (Ratio) | -60.78 | 55.85 | -85.71 | 5.21 | 107.34 |
|  | [-618.61,497.05] | [-163.29,274.98] | [-373.48,202.07] | [-313.58,324.00] | [-80.95,295.62] |
| Advocacy (Ratio) | -15.55 | -6.93 | 8.01 | -7.00 | 2.47 |
|  | [-48.72,17.63] | [-19.96,6.10] | [-9.10,25.13] | [-25.95,11.96] | [-8.73,13.67] |
| Pre-admission C(E)TR (Ratio) | -18.02 | -1.52 | -13.78 | 0.80 | -10.10 |
|  | [-95.59,59.56] | [-32.00,28.95] | [-53.80,26.24] | [-43.53,45.13] | [-36.28,16.08] |
| Post-admission C(E)TR (Ratio) | 86.40 | 16.96 | 0.40 | -37.27 | -11.23 |
|  | [-57.46,230.26] | [-39.55,73.47] | [-73.81,74.62] | [-119.48,44.95] | [-59.79,37.32] |
| Local Authority Aware (Ratio) | **-71.11 *** | 18.88 | -10.24 | 11.28 | **25.33 *** |
|  | [-134.14,-8.07] | [-5.88,43.65] | [-42.76,22.28] | [-24.75,47.30] | [4.06,46.61] |
| Discharge Destination (Ratio) | -6.20 | **-4.42 *** | -2.58 | -0.00 | 0.00 |
|  | [-14.81,2.40] | [-7.80,-1.04] | [-7.02,1.85] | [-4.92,4.91] | [-2.90,2.91] |
| N | 64 | 64 | 64 | 64 | 64 |
| AIC | 650.90 | 531.30 | 566.18 | 579.28 | 511.88 |
